# Prevalence and predictors of slow coronary flow phenomenon in Kermanshah province

**DOI:** 10.34172/jcvtr.2021.03

**Published:** 2021-01-13

**Authors:** Mohammad Rouzbahani, Saeid Farajolahi, Nafiseh Montazeri, Parisa Janjani, Nahid Salehi, Alireza Rai, Reza Heidari Moghadam, Arsalan Naderipour, Asal Kanjorpor, Etrat Javadirad, Javad Azimivghar

**Affiliations:** ^1^Cardiovascular Research Center, Health Institute, Kermanshah University of Medical Sciences, Kermanshah, Iran; ^2^Department of Emergency Medicine, School of Paramedics, Kermanshah University of Medical Sciences, Kermanshah, Iran; ^3^Clinical Research Development Center of Imam Khomeini Hospital, Kermanshah University of Medical Sciences, Kermanshah, Iran

**Keywords:** Coronary Angiography, Slow Coronary Flow Phenomenon, Predictor, Prevalence

## Abstract

***Introduction:*** This study was conducted to investigate prevalence and predictors of slow coronary flow phenomenon (SCF) phenomenon.

***Methods:*** This cross-sectional study was performed at Imam Ali Cardiovascular Hospital affiliated with the Kermanshah University of Medical Sciences (KUMS), Kermanshah province, Iran. From March 2017 to March 2019, all the patients who underwent coronary angiography were enrolled in this study. Data were obtained using a checklist developed based on the study’s aims. Independent samples *t* tests and chi- square test (or Fisher exact test) were used to assess the differences between subgroups. Multiple logistic regression model was applied to evaluate independent predictors of SCF phenomenon.

***Results:*** In this study, 172 (1.43%) patients with SCF phenomenon were identified. Patients with SCF were more likely to be obese (27.58±3.28 vs. 24.12±3.26, *P* <0.001), hyperlipidemic (44.2 vs. 31.7, *P* <0.001), hypertensive (53.5 vs. 39.1, *P* <0.001), and smoker (37.2 vs. 27.2, *P* =0.006). Mean ejection fraction (EF) (51.91±6.33 vs. 55.15±9.64, *P* <0.001) was significantly lower in the patients with SCF compared to the healthy controls with normal epicardial coronary arteries. Mean level of serum triglycerides (162.26±45.94 vs. 145.29±35.62, *P* <0.001) was significantly higher in the patients with SCF. Left anterior descending artery was the most common involved coronary artery (n = 159, 92.4%), followed by left circumflex artery (n = 50, 29.1%) and right coronary artery (n = 47, 27.4%). Body mass index (BMI) (OR 1.78, 95% CI 1.04-2.15, *P* <0.001) and hypertension (OR 1.59, CI 1.30-5.67, *P* =0.003) were independent predictors of SCF phenomenon.

***Conclusion:*** The prevalence of SCF in our study was not different from the most other previous reports. BMI and hypertension independently predicted the presence of SCF phenomenon.

## Introduction

Slow coronary flow (SCF phenomenon) phenomenon was firstly described by Tambe et al in 1972 ^1^. SCF phenomenon phenomenon is characterized by the delayed distal vessel opacification of contrast, without occlusion. So that, in the patients with chest pain undergoing coronary angiography, actually, a delayed progression of the contrast material is found through epicardial coronary arteries without obstruction.^2^ Thrombolysis in Myocardial Infarction (TIMI) frame count (TFC) is used to assess speed of contrast materials progression in the coronary arteries.^3^

SCF phenomenon is not a frequent finding in routine coronary angiography. It has been reported that 1%–5% of the patients undergoing coronary angiography are suffering from SCF phenomenon.^4,5^ SCF phenomenon is more commonly found in young men and current smokers having chest pain.^4^ SCF phenomenon involves both small and epicardial coronary arteries and has been suggested as an early phase of atherosclerosis.^6,7^

Pathophysiology of SCF phenomenon is not completely known. Although, endothelial dysfunction, diffuse atherosclerosis, and inflammation are significantly associated with pathogenesis of SCF phenomenon.^4,8,9^ Previous studies have reported different clinical risk factors to be independently related to SCF phenomenon .^4,10-13^

Given the existence of poor documents regarding pathophysiological mechanism of SCF phenomenon, there is a need to determine the main risk factors of SCF. Until now, no study has been done to investigate clinical risk factors related to SCF phenomenon on our study population, thus the current study was undertaken to address this research gap.

Therefore, this study was conducted to investigate angiographic prevalence and clinical predictors of SCF phenomenon in the patients who underwent coronary angiography at Imam Ali Cardiovascular Hospital affiliated with the Kermanshah University of Medical Sciences (KUMS), Kermanshah province, Iran from March 2016 to March 2018.

## Materials and Methods

### Study Population and Design

This cross-sectional study was performed at Imam Ali Cardiovascular Hospital, affiliated with KUMS, Kermanshah province, Iran. From March 20, 2017 to March 20, 2019, all the patients who underwent coronary angiography were assessed for inclusion in this study. Patients aged ≥18 years old presenting with normal epicardial coronary arteries (NECA) but having SCF on angiogram were selected (n=172). Also, patients aged ≥18 years old presenting with NECA and having normal flow on angiogram were selected (n=1848). SCF phenomenon is characterized by the delayed distal vessel opacification of contrast, in the absence of significant epicardial coronary stenosis. So that, in the patients with chest pain undergoing coronary angiography, actually, a delayed progression of the contrast material is found through epicardial coronary arteries without obstruction.^2^ Angiographic film should be prepared at a speed of 30 frames per second and contrast injection should be done by a 6F catheter to measure TFC. In the first frame, contrast material fully opacifies origin of the artery. The last frame is predefined for each coronary artery: so that, for the left anterior descending (LAD) and circumflex (Cx) arteries, it shows the most distal bifurcation, whereas for right coronary artery (RCA), it shows emergence of the first posterolateral (PL) branch. For LAD, apical segment is the milestone for TFC. Because, LAD is usually longer than the other arteries, a correction factor is required when calculating this score by dividing TFC of LAD by 1.7. Cut point of TFC was equal to 21 ± 2 for LAD, and it was equal to 22 ± 4 and 20 ± 3 for LCx and RCA, respectively.^14^ Patients who had coronary artery diseases (such as plaque, spasm, ectasia, stenosis, or obstructive lesion), and/or they had previously undergone coronary artery bypass grafting (CABG) or percutaneous coronary intervention (PCI), and/or they had embolism, heart failure, valvular heart disease, and connective tissue disorders, as well as those who were not resident in city of Kermanshah (living for less than 6 months), and those with incomplete personal or medical information, were excluded from the study.

### Instruments and Data Collection

Data were collected by a nurse who was well trained in data collection using a checklist developed based on the study’s aims. He extracted the data from the patients’ medical records (including both paper and electronic medical records). The checklist was assessed and approved by obtaining experts҆ opinions including a statistician and two cardiologists. All the checklists were checked and verified by a general physician who was responsible for quality control. The checklist was comprised of five following parts: demographic characteristics (e.g., gender), clinical histories (e.g., diabetes mellitus), laboratory parameters (e.g., C-reactive protein (CRP)), angiographic findings (e.g., culprit vessels), and electrocardiography data.

### Statistical analysis

Statistical analysis was performed by the statistical package for social sciences (SPSS) statistical software (Version 23.0; IBM Corporation, Chicago, USA). Quantitative variables (e.g., age) were described using mean ± standard deviation (SD) and qualitative/categorical variables (e.g., smoking) were expressed as frequencies and percentages. Differences between groups were evaluated using the Chi-square test (or Fisher exact test) for categorical variables, and independent samples*t* test for continuous and normally distributed variables. For assessing independent predictors of SCF phenomenon, multiple logistic regression model was applied. In logistic regression analysis, variables with *P* <0.20 were entered in bivariate analysis. Odds ratios (ORs) and 95% confidence intervals (CIs) were calculated for all the variables. All the analyses were considered to be significant at *P* < 0.05.

## Results

A total of 11 970 coronary angiographies were performed at Imam Ali Cardiovascular Hospital from March 2016 to March 2019, as a result of which 1 848 (15.43%) patients with NECA and 172 (1.43%) patients with SCF phenomenon were identified. Demographic and clinical characteristics of the patients are reported in Table 1. Mean age of the patients with SCF was equal to 53.07 ± 9.81 years old, and for the patients with NECA, it was equal to 52.17 ± 10.84 years old (*P* = 0.256). Mean BMI was equal to 27.58 ± 3.28 for the patients with SCF vs. 24.12 ± 3.26 for the patients with NECA (*P* <0.001). Prevalence rates of the current smoker (37.2% vs. 27.2%, *P* = 0.006), hypertension (53.5% vs. 39.1%, *P* < 0.001), and hypercholesterolemia (44.2% vs. 31.7, *P* < 0.001) were significantly higher in the patients with SCF compared to the patients with NECA. Of course, blood pressure of >139/89 mm Hg was defined as hypertension ^15^. Patients with NECA were more likely to have negative C-reactive protein (80.5% vs. 51.2%, *P* < 0.001). Comparing the lipid profiles, it was observed that, mean level of serum triglycerides (162.26 ± 45.94 vs. 145.29 ± 35.62, *P* < 0.001) was significantly higher in the patients with SCF compared to the patients with NECA. Mean EF (51.91 ± 6.33 vs. 55.15 ± 9.64, *P* < 0.001) was significantly lower in the patients with SCF compared to the patients with NECA (Table 1).

**Table 1:** Demographic and clinical characteristics of patients (n=2020)

**Variable**	**SCF (n= 172)**	**NECA (n=1848)**	***P*** ** value**
Age, y	53.07 ± 9.81	52.17 ± 10.84	0.256*
Body mass index, kg/m^2^	27.58 ± 3.28	24.12 ± 3.26	<0.001*
Male	121 (70.3)	1310 (70.9)	0.882**
Current smoker	64 (37.2)	503 (27.2)	0.006**
Diabetes mellitus	53 (30.8)	470 (25.4)	0.123**
Hypertension	92 (53.5)	723 (39.1)	<0.001**
Hypercholesterolemia	76 (44.2)	586 (31.7)	<0.001**
Hematocrit	45.69 ± 19.17	46.89 ± 17.67	0.430*
Platelet^¶^	222000.94 ± 62000.23	220000.89 ± 61000.67	0.686*
Erythrocyte sedimentation rate	14.16 ± 9.91	13.69 ± 9.17	0.550*
C-reactive protein			
Negative	88 (51.2)	1488 (80.5)	
1^+^	60 (34.9)	355 (19.2)	<0.001***
2^+^	23 (13.4)	5 (0.3)	
3^+^	1 (0.6)	0 (0)	
Low-density lipoprotein	118.89 ± 41.67	116.87 ± 38.15	0.541*
High-density lipoprotein	44.36 ± 9.09	45.14 ± 9.83	0.286*
Triglycerides	162.26 ± 45.94	145.29 ± 35.62	<0.001*
Ejection fraction	51.91 ± 6.33	55.15 ± 9.64	<0.001*

Abbreviations: SCF, slow coronary flow; NECA, normal epicardial coronary arteries Continuous variables expressed as mean ± SD, otherwise n (%) *Independent samples *t* -test; ** Chi-square; *** Fisher exact test

Out of 172 patients with SCF, 111 (64.5%) of them had stable angina, 49 (28.5%) of them had unstable angina, 5 (2.9%) of them had ST-segment elevation myocardial infarction (STEMI), 3 (1.7%) of them had non-STEMI, and 4 (2.3%) of them had ventricular tachycardia. Out of 172 patients with SCF, 98 (57.0%) of them had normal electrocardiography, 69 (40.1%) of them presented ST-T change, and 5 (2.9%) of them presented STEMI.

**Figure 1:**
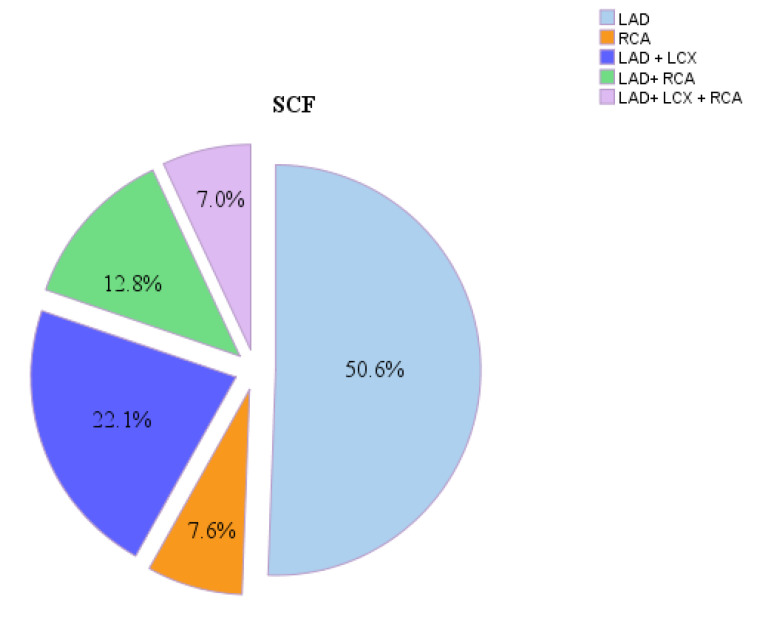
Slow flow pattern (numbers of culprit vessels) in our study population(n = 172)

Out of 172 patients with SCF, 100 (58.2%) of them had slow flow in 1 artery, 60 (34.9%) of them had slow flow in 2 arteries, and 12 (7.0%) of them had slow flow in all 3 arteries. The most common involved artery was LAD (n = 159, 92.4%), followed by LCx (n = 50, 29.1%) and RCA (n = 47, 27.4%) (Figure 1).

Mean TFC in LAD (39.60 ± 4.51 vs. 17.79 ± 2.84, *P* < 0.001), LCx (40.41 ± 3.86 vs. 16.99 ± 1.83, *P* < 0.001), and RCA (36.69 + 3.31 vs. 18.69 + 2.34, *P* < 0.001 indicated slow flow phenomenon in the patients with SCF (Table 2).

**Table 2:** TIMI frame count in patients with SCF versus NECA

**Variable**	**SCF** **Mean **± **SD**	**NECA** **Mean **± **SD**	***P *** **value**
LAD	39.60 ± 4.51	17.79 ± 2.84	< 0.001*
LCX	40.41 ± 3.86	16.99 ± 1.83	< 0.001*
RCA	36.69 ± 3.31	18.69 ± 2.34	< 0.001*

Abbreviations: SCF, slow coronary flow; NECA, normal epicardial coronary arteries; SD, standard deviation; LAD, left anterior descending artery; LCx, left circumflex artery; RCA, right coronary artery *Independent samples *t* test

Analysis of the results of multiple logistic regression identified BMI (OR 1.78, 95% CI 1.04-2.15, *P* < 0.001) and hypertension (OR 1.59, CI 1.30-5.67, *P* = 0.003) as independent predictors of SCF phenomenon (Table 3).

**Table 3:** Independent predictors of the SCF

**Predictors**	**OR (95% CI)**	***P*** value*
Body mass index	1.78 (1.04-2.15)	<0.001
Current smoker	1.01 (0.98-1.88)	0.093
Diabetes mellitus	0.54 (0.30-3.32)	0.437
Hypercholesterolemia	0.76 (0.14-1.71)	0.233
Hypertension	1.59 (1.30-5.67)	0.003
Triglycerides	0.65 (0.47-3.65)	0.763

Abbreviations: SCF, slow coronary flow; OR, odd ratio; CI, confidence interval *Multiple logistic regression

## Discussion

This cross-sectional study was performed on the patients who underwent coronary angiography at Imam Ali Cardiovascular Hospital affiliated with KUMS, Kermanshah province, Iran from March 2017 to March 2019, to determine angiographic prevalence and clinical predictors of SCF phenomenon. To the best of our knowledge, the present study was the largest study particularly investigated clinical features of the patients with SCF phenomenon in an Iranian population in west of the country. Strength of the present study was its large sample size compared to the other studies assessing SCF phenomenon.

In our study, prevalence of SCF phenomenon was determined as 1.43%. Sanati et al, reported a prevalence of about 2% in a study from Iran in 2016.^16^ Hawkins et al in a study from Oklahoma, USA found a prevalence rate of 5.5% in the patients who underwent coronary angiography in 2012.^17^ Beltrame et al reported that 1% of all the patients who underwent coronary angiography had SCF phenomenon in Australia.^4^ In 2018, Mukhopadhyay found that prevalence rate of SCF phenomenon was equal to 0.8% in India.^18^ The reasons for these subtle differences are not clearly known; however, discrepancy in atherosclerotic burdens and cardiovascular risk factors among different ethnic populations may clarify these differences. SCF phenomenon has been suggested as an early phase of atherosclerosis, and is manifested by micro vascular dysfunction.^19^ Finally, differences in ethnic background, atherosclerotic burdens, and related comorbidities of the studied populations might explain these discrepancies.

Our results demonstrated that the patients with SCF were more plausible to be obese, hyperlipidemic, hypertensive, and smoker. Ghaffari et al indicated that the patients with SCF were more plausible to be obese and active smoker.^20^ Sanghvi et al found a higher prevalence of hypertension, dyslipidemia, and smoking in the patients with SCF.^21^ Hawkins et al found that the patients with SCF were more obese.^17^ Sanati et al reported that the patients with SCF were more likely to be hypertensive compared to those with NECA.^16^ Moazenzadeh et al reported that incidence of systolic hypertension was significantly higher in the SCF group compared to control group.^13^ Yaron Arbel et al showed that the patients with SCF were more plausible to be smoker compared to those with normal coronary flow.^22^ Moreover, as shown in the study by Xia et al herein, it was found that the patients with SCF had a higher level of CRP.^12^ CRP, as a susceptible marker of systemic inﬂammation is an important predictor of cardiovascular diseases. In agreement with our study, Ghaffari et al reported that the patients with SCF had a higher level of triglyceride compared to the patients with normal coronary flow.^20^ Our results demonstrated that mean EF was significantly lower in the patients with SCF compared to the NECA group. These results are consistent with the findings of the study by Sanati et al who showed that mean EF was significantly lower in the patients with SCF compared to NECA group.^16^

Besides, it was found that BMI and hypertension were independent predictors of SCF phenomenon, indicating that the patients with higher BMI and hypertension are at higher risk for development of SCF phenomenon. In line with our finding, a study conducted on Iranian population also introduced hypertension as an independent predictor of SCF phenomenon.^13^ Sanghvi et al in a study from India reported hypertension as the strongest predictor of SCF phenomenon in 2018.^21^ Hawkins et al observed that higher BMI independently predicted the presence of SCF phenomenon.^17^ Yilmaz et al evaluated clinical features of SCF phenomenon in a Turkish population, and identified BMI as independent predictor of SCF phenomenon.^11^ Chaudhry et al indicated that higher BMI independently predicted SCF phenomenon.^23^ Conversely, Sanati et al showed lower BMI as independent predictor of SCF phenomenon.^16^ Pathophysiology of SCF phenomenon is not completely known, however, the literature supports endothelial dysfunction and links to atherosclerosis as documenting evidence. Actually, indirect evidence proposes that obesity might be related to endothelial dysfunction, consequently leading to development of SCF phenomenon. Besides, Wannamethee et al assessed 4,000 elderly men and found a strong relationship between BMI and markers of endothelial dysfunction.^24^

Moreover, our results showed that the most common involved artery was LAD with a rate of 92% followed by LCx and RCA. This result is in accordance with the findings of the study by Sanati et al who reported LAD as the most common involved artery, with a rate exceeding 90%.^16^ Our finding was also in line with a previous study by Sanghvi et al in which LAD (82.5%) was the most common involved artery followed by LCx artery (67.5%) and RCA (60%).^21^ Furthermore, Beltrame et al indicated LAD as the most common involved artery in 86% of the patients with SCF.^19^ The artery involvement reported in the present study varied from that of the other studies. Hawkins et al found that LAD, LCx, and RCA were involved in 67, 69, and 58% of the patients , respectively.^17^ The reason for this discrepancy is unknown, though it may be due to racial differences and technical errors.

Clinical presentation of SCF phenomenon is diverse ranging from stable or unstable angina and NSTEMI to STEMI.^25,26^ In the present study, 64.5% of the patients with SCF presented with stable angina and remaining of them (33.2%) presented with acute coronary syndrome (ACS) (28.5% with unstable angina, 2.9% with STEMI, and 1.7% with NSTEMI). Mukhopadhyay in a study from India in 2018 reported that 50% of the patients with SCF presented with stable angina and 50% of them presented with ACS (35% with unstable angina and 15% with NSTEMI).^18^ Likewise, in an earlier study done on Iranian population, it was reported that 75% of the patients with SCF presented with ACS.^16^ Sanghvi et al found that ACS (42.5%) was the most common clinical presentation in the patients with SCF.^21^ Beltrame et al reported that 75% of the patients with SCF phenomenon presented with ACS.^4^ Moreover, Yaron Arbel et al in a study from Israel reported that non-specific chest pain (71.9%), ACS (18.4%) and stable angina (8.8%) were among the most common presenting complaints in the patients with SCF, respectively.^22^

Our study had several limitations. Firstly, cross-sectional nature of the present study did not allow further evaluation of any apparent associations over time; hence for evaluating causality, longitudinal studies with an extended follow-up should be done. Secondly, our data were obtained from a single center; therefore, our participants may not be representative of the whole patients with SCF phenomenon. Moreover, the patients’ usage of medication was not reported in this study.

## Conclusion

Our results showed that in the studied population, prevalence rate of SCF phenomenon was equal to 1.43%. Also, BMI and hypertension independently predicted the presence of SCF phenomenon. The most common involved artery was LAD followed by LCx and RCA. Majority of the patients with SCF presented with stable angina. Accordingly, further studies are needed to determine mechanisms of action of the mentioned predictors (BMI and hypertension). Finally, the current study provides a foundation for future studies that should be conducted in the other ethnic groups residing in different parts of Iran.

## Acknowledgments

The authors would like to thank the Kermanshah University of Medical Sciences (KUMS) for funding this project. Also, the authors greatly appreciate the staff working at the Imam Ali Cardiovascular Hospital, Kermanshah province, Iran.

## Competing interest

The authors declare that they have no conflict of interest. In addition, the authors have no financial interest related to any aspect of the study.

## Ethical approval

The study protocol and research process were approved and monitored by the Research Ethics Committee at KUMS (IR.KUMS.REC.1398.380). Moreover, the individuals҆ personal information was kept confidential with the access limited to researcher.

## Funding

This research project was funded by the Kermanshah University of Medical Sciences (funding number=980588).
